# OP69 EuroQol Health And Wellbeing Short Version Validation In A Brazilian Social Program: Psychometric Properties And Applicability In Vulnerable Populations

**DOI:** 10.1017/S0266462325101268

**Published:** 2025-12-29

**Authors:** Ana Helena Santos, Andréa Monteiro, Marisa Santos, José Evangelista, Daniel Gigliotti

## Abstract

**Introduction:**

Instituto Dara, a Brazilian non-profit organization, supports vulnerable families through multidimensional interventions addressing poverty and chronic illness. While validated quality-of-life instruments are essential for program evaluation, few target disadvantaged populations with chronically ill children in Brazil. This study evaluated the psychometric properties of the EuroQol Health and Wellbeing Short Version (EQ-HWB-S) questionnaire, including its convergent validity and test-retest reliability.

**Methods:**

The study included 99 individuals representing families supported by Instituto Dara. Data collection used face-to-face interviews with EQ-HWB-S and additional validated instruments (EuroQol-5D and the Warwick-Edinburgh Mental Wellbeing Scale [WEMWBS]) to assess convergent validity. Test-retest reliability was assessed in 45 participants over a two-week period. Statistical analyses included descriptive methods and correlation analyses to summarize EQ-HWB-S applicability and performance in this population.

**Results:**

Physical domains (mobility and activities) showed minimal impairment, with most participants at level one (>75%). In contrast, psychosocial domains showed significant burden: over 50 percent reported moderate to extreme levels of exhaustion, loneliness, and anxiety or depression. Test-retest reliability showed moderate agreement (r=0.568; p<0.001). The EQ-HWB-S yielded an overall score of 0.707. Construct validity analyses demonstrated moderate correlations with established measures (r=0.33 to 0.45), particularly with the physical well-being domains of the EQ-5D and alignment with the WEMWBS for psychological dimensions.

**Conclusions:**

The results highlighted the significance of EQ-HWB-S in assessing domains such as loneliness, exhaustion, and control. The instrument demonstrated robust psychometric properties with moderate test-retest reliability and strong convergent validity, though its discriminatory ability warrants further investigation. These findings support its use for assessing quality-of-life outcomes in vulnerable populations with chronically ill children.

